# How one block of trials influences the next: persistent effects of disease prevalence and feedback on decisions about images of skin lesions in a large online study

**DOI:** 10.1186/s41235-022-00362-0

**Published:** 2022-02-02

**Authors:** Jeremy M. Wolfe

**Affiliations:** 1 Visual Attention Lab, Department of Surgery, Brigham and Women’s Hospital, 900 Commonwealth Ave, 3rd Floor, Boston, MA 02215 USA; 2 Harvard Medical School, Boston, USA

## Abstract

Using an online, medical image labeling app, 803 individuals rated images of skin lesions as either "melanoma" (skin cancer) or "nevus" (a skin mole). Each block consisted of 80 images. Blocks could have high (50%) or low (20%) target prevalence and could provide full, accurate feedback or no feedback. As in prior work, with feedback, decision criteria were more conservative at low prevalence than at high prevalence and resulted in more miss errors. Without feedback, this low prevalence effect was reversed (albeit, not significantly). Participants could participate in up to four different conditions a day on each of 6 days. Our main interest was in the effect of Block *N* on Block *N* + 1. Low prevalence with feedback made participants more conservative on a subsequent block. High prevalence with feedback made participants more liberal on a subsequent block. Conditions with no feedback had no significant impact on the subsequent block. The delay between Blocks 1 and 2 had no significant effect. The effect on the second half of Block 2 was just as large as on the first half. Medical expertise (over the range available in the study) had no impact on these effects, though medical students were better at the task than other groups. Overall, these seem to be robust effects where feedback may be 'teaching' participants how to respond in the future. This might have application in, for example, training or re-training situations.

## Introduction

In visual decisions about finding and/or identifying a target, the prevalence of the target makes a difference (Horowitz, 2017). By “prevalence,” we mean the frequency with which a target appears in a series of trials or cases. The effects of prevalence are of more than academic interest because target prevalence can vary dramatically across tasks in the real world. For example, in a task like identifying signs of breast cancer in mammographic images, the prevalence is very low in a breast cancer screening program where cancer might be present on 0.5% of images and where findings that are suspicious enough to require more testing might be present on 5–10% of cases (e.g., Jackson et al., 2012). The prevalence of a disease will be much higher in a set of images referred to the radiologist because an initial screening was suspicious. The classic low prevalence effect (LPE) involves an increase in false negative/miss errors and, usually, a decrease in the rate of false positive/false alarm errors (Wolfe et al., 2005, 2007). In signal detection terms, the LPE can be described largely, but not entirely, as a “criterion shift” in which participants become more ‘conservative’ about declaring a target to be present (Hautus et al., 2021). In visual search studies of the LPE, participants also tend to abandon search more quickly when targets are not found (Wolfe & Van Wert, 2010).

We also know that what you have seen influences what you will report seeing next, for example, in classic adaptation effects (e.g., Helson, 1964) or in serial dependence effects like those studied by Fischer and Whitney (2014) and many others (e.g., Gekas et al., 2019 or Manassi et al., 2021 for work with radiologists). What you are *told* that you will see also has an impact. Thus, you will look harder if you are given information thatsuggests that it is likely that there is something to find (Reed et al., 2014; Littlefair et al., 2016). In this paper, we examine these effects together. What is the effect of the prevalence in one block on performance on a subsequent block?

Our understanding of prevalence effects was complicated in 2018, when Levari et al. (2018) reported that it was possible to obtain effects in the opposite direction from the classic LPE. Their participants made decisions about a single item on a continuum. For instance, participants might be asked if a dot, drawn from a blue-purple color continuum, was ‘blue.’ When fewer dots were drawn from the blue end of the continuum, they reported that participants became more liberal about calling ambiguous dots ‘blue.’ They called this effect “prevalence-induced concept change” (PICC). Lyu et al. (2021) found that one driver of these opposing LPE and PICC results was the presence or absence of feedback. When making decisions about the same stimulus continuum, participants reliably produced LPE effects when given feedback after each trial. They tended to produce PICC effects (somewhat less reliably) when there was no feedback. This has real-world implications because, just as tasks differ in target prevalence, they differ in feedback. For instance, in training, participants might receive immediate feedback after every trial. In the field, that feedback might be delayed, partial, or unavailable. Consider airport security screening. In training, participants are likely to see targets at relatively high prevalence, with feedback. At the airport checkpoint, real ‘threats’ will be rare (we may hope!) and security screeners may get some feedback about some positive cases because the suspect bag is opened on the spot. False negative errors probably generate no feedback, even though these would be the most serious errors in a security setting.

The central question of the present study is how experience with one level of prevalence, with or without feedback, influences performance on subsequent trials where either prevalence and/or feedback conditions could have changed. In the great bulk of research on prevalence effects, high prevalence trials were followed by low prevalence trials or participants experienced only a single prevalence level. Typically, feedback is not independently manipulated (though see Growns & Kukucka, 2021; Lyu et al., 2021; Papesh et al., 2018; Weatherford et al., 2020). Moreover, in previous studies, the change due to a previous discrete block of trials was not assessed.

In the present study, prevalence can be high or low and feedback can be present or absent. This yields four conditions: low prevalence with feedback, low prevalence without feedback, high prevalence with feedback, and high prevalence without feedback. There are 16 possible pairings of two consecutive blocks. In this study, the stimuli are skin lesions: either nevi (skin 'moles'; singular, nevus) or melanoma (skin cancer). A large dataset of over 300,000 decisions was collected online by participants using a medical image labeling app (“DiagnosUs” http://diagnosus.com/). Participants had varying levels of expertise from complete novice to MD as will be described later. Thus, in addition to allowing us to assess the influence of one prevalence X feedback combination on another, these data provide new evidence about prevalence effects in expert populations (Evans et al., 2011, 2013; Evered, 2017; Mitroff et al., 2017; Reed et al., 2011; Trueblood et al., 2021; Wolfe et al., 2013).

To anticipate the broad outlines of the results, LPE and weak PICC effects are produced with these dermatology stimuli. As in other studies with expert populations, our experts show these effects, as do novices. In terms of the influence of one block of trials on the next block, we find that, in general, the experience with a low prevalence block with feedback makes one more conservative in any subsequent block while the experience of high prevalence with feedback makes one more liberal. A block without feedback does not appear to have a significant impact on the next block.

## Methods

**Fig. 1:**
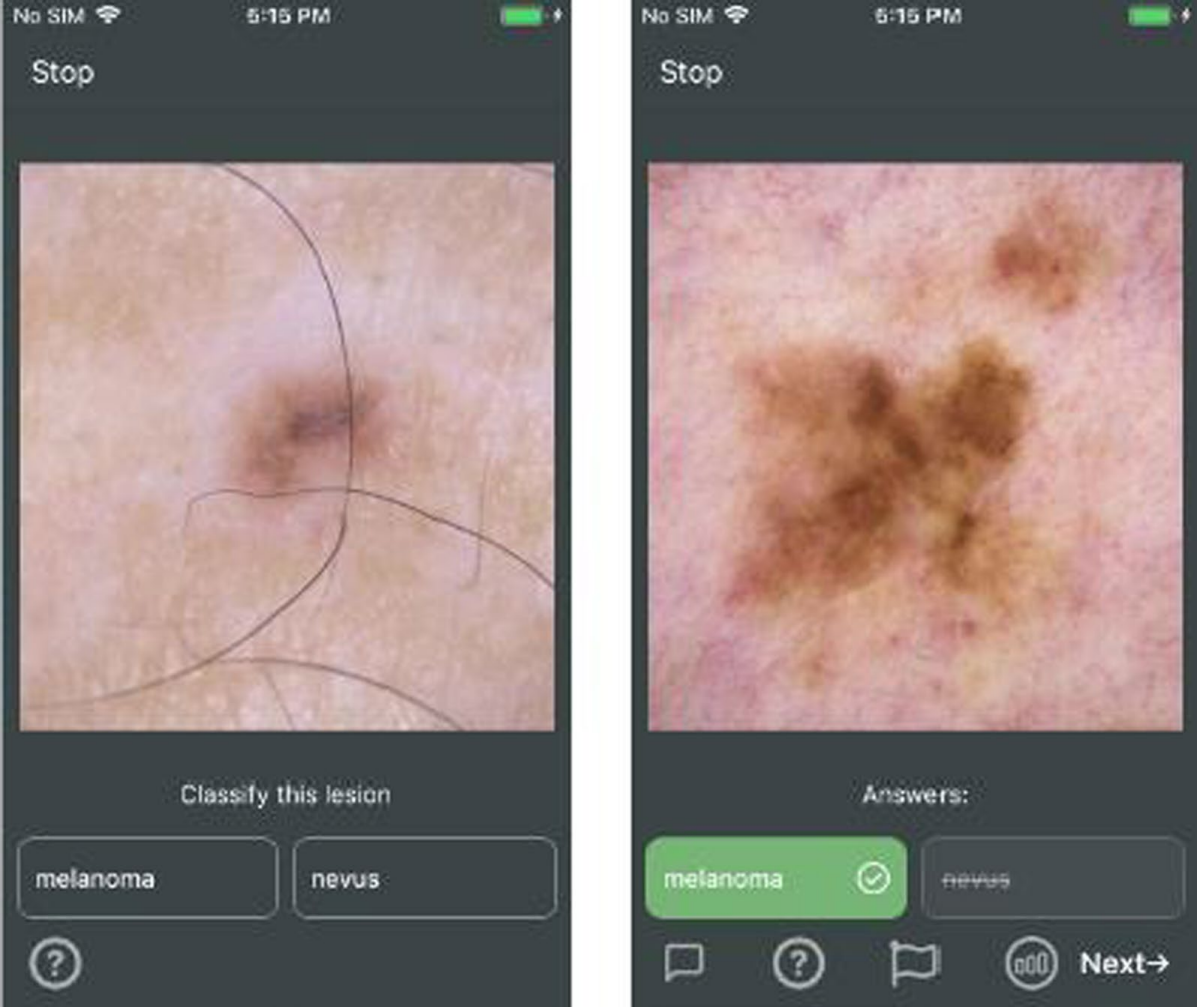
Sample stimulus displays. Left: participants are asked if a lesion is a melanoma (cancer) or a nevus (benign ‘mole’). Right: after response, if this were a feedback condition, red or green feedback would inform the participant of the correctness of the answer. In no feedback conditions, neutral feedback indicated that the response had been registered

Data were collected online via a free and open iOS application, “DiagnosUs,” created by Centaur Labs as a platform for game-like image labeling competitions ( https://www.centaurlabs.com/). Participants can win cash prizes for rising to the top of the leaderboard in these competitions. Between 6/22/21 and 6/27/21, Centaur Labs ran 24 ‘contests’ on our behalf, collecting > 300,000 trials from 803 participants in 6 days. Each of the 24 contests consisted of 80 unique images of a skin lesion as shown in Fig. 1. This is a relatively small number of images, but it kept the sessions short and encouraged participation. Images came from the International Skin Imaging Collaboration (ISIC) 2018 challenge (Codella et al., 2019). The set contains over 1200 melanoma images and over 7600 nevus images.

On each of the 6 days, there were four contests available. These were:

1. *Low prevalence* No feedback; consisting of 20% (16) melanoma images
2. *Low prevalence* Feedback; prevalence was the same as in #1, but with accurate, trial-by-trial feedback
3. *High prevalence* No feedback; consisting of 50% (40) melanoma images
4. *High prevalence* Feedback; prevalence was the same as in #3, but with accurate, trial-by-trial feedback

The contests were randomly assigned to participants with the constraint that they did not run the same type of contest more than once in a day. Participants could take part in as many or as few contests as they wished over the course of the 6 days and they could end up repeating a condition from 1 day on another day. The prevalence and feedback conditions were not advertised to participants. Accuracy is the main dependent variable though we also collected response times.

Because of the online, voluntary nature of the data collection, we did not have control over the viewing conditions or the type of screen used. We compensated by collecting a very large dataset (see below).

### Observers

We collected data from 803 unique individuals. For each individual, we have a crude categorization of expertise as shown in Table 1:

**Table 1:** Observers divided by expertise category

Medical student	337
Pre-med student	174
No medical experience	145
Medical doctor	54
Other healthcare professional	47
Nurse	25
Medical technician	11
Physician assistant	10

Participants were asked why they chose to participate and given three choices, as shown in Table 2.

**Table 2:** Observers divided by reason to participate

Compete with others	37
Earn money	248
Improve my skills	518

Participants reported coming from 78(!) countries with three quarters of the participants coming from nine countries, as shown in Table 3.

**Table 3:** Reported country of origin

Philippines	198
USA	182
Ghana	94
Great Britain	31
Romania	30
Canada	27
Mexico	19
Indonesia	15
Australia	13
All other	194

Beyond this demographic information, we have only an observer number. We were given no identifying information about the participants. Participation on the app constituted consent. Procedures were approved by the Institutional Review Board at Brigham and Women’s Hospital (IRB #2007P000646).

These 803 participants produced 311,842 trials of data. An excel spreadsheet with all data is posted on the Open Science Framework at https://osf.io/hck5n/. From this set, we eliminated participants who did not complete a full block of 80 trials. This left 630 participants who ran a total of 277,371 trials in 2988 blocks. Different participants chose to participate in different numbers of blocks, as shown in Table 4.

**Table 4:** Numbers of participants who ran *N* (1–24) blocks over 6 days of testing

Blocks run	# of participants	Blocks run	# of participants
1	315	13	10
2	178	14	2
3	104	15	4
4	56	16	6
5	52	17	5
6	22	18	3
7	27	19	10
8	20	20	3
9	14	21	4
10	10	22	2
11	10	23	6
12	5	24	4

Those participants running only a single block were removed from most analyses because our primary interest is the influence of one block upon the next. Obviously, with some participants contributing 24 blocks and others contributing 2, the dataset is unbalanced. We repeated the main analyses reported below, limiting the analysis to only the first pair of blocks for each observer. This reduces power. However, the main patterns of results, reported below, are found when the dataset is restricted to only blocks 1 and 2. Accordingly, we think that the unequal numbers of trials and blocks from different participants are not a significant issue for the present study. In the results reported below, we analyze results from the 2998 remaining blocks of data.

## Results

To analyze the effect of one block on the next block, we derived all the pairs of blocks in the dataset. For each of the blocks, we calculated the true positive and false positive proportions and used these to derive the standard signal detection measure of *d*′ and criterion (Note that we are refraining from using the term “sensitivity” because it is used to refer to *d*′ in the behavioral science community and to the true positive rate in the medical community). For the primary analyses, pairs were removed from analysis if either member of the pair produced a *d*′ of less than 0.5. The task was relatively difficult and we had little control over the motivation of participants in online study of this sort. The probability of achieving a *d*′ > 0.5 by guessing through an 80 trial block is ~ 0.6%. A cutoff of 0.4 would permit over 2% of blocks to be pure guessing. A *d*′ > 0.5 cutoff left 2080 pairs of blocks. The distribution of those pairs is shown in Table 5.

**Table 5:**
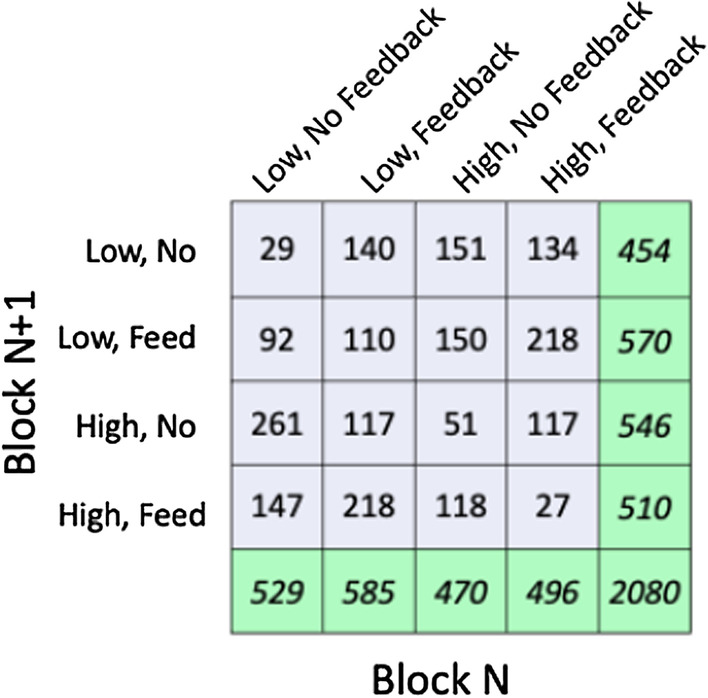
Counts of the 16 different pairs of Block *N* and Block *N* + 1

As can be seen, there is a good, if uneven distribution of pairs. There are fewer pairs on the diagonal where the condition is the same in Blocks *N* and *N* + 1. In order to run the same block, twice in a row, participants needed to encounter that condition last on Day *K* and first on Day *K* + 1 since the same condition could not be run twice on the same day. The marginals show that this distribution of the four different conditions is very roughly uniform.

**Fig. 2:**
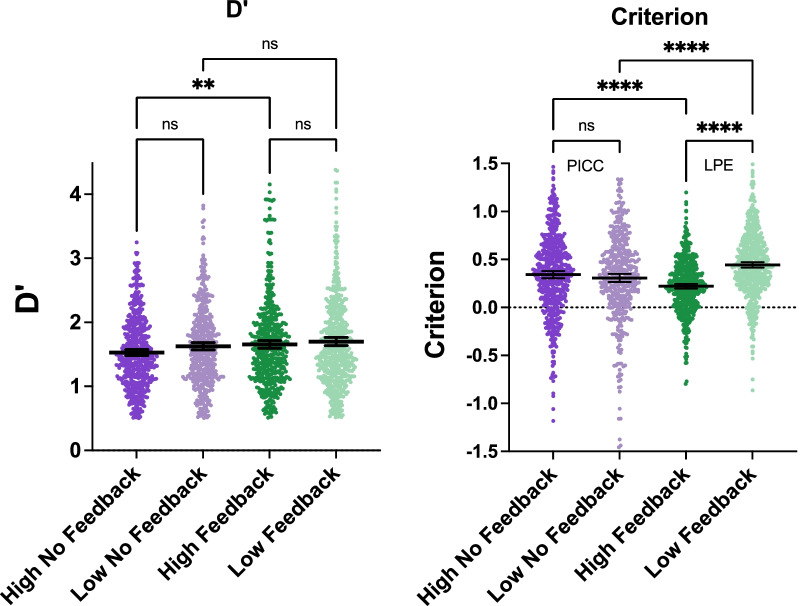
*D*′ and criterion as a function of condition. Each dot represents one of 2080 blocks of data. Black lines show mean and ± 95% CI of the mean

First, we compare performance in the four conditions, regardless of their position the sequence of sessions for each observer. Figure 2 shows the signal detection measures of *d*′ and criterion, “*c*.” These values cannot be computed if *p*(True Positive) = 1.0 or *p*(False Positive) = 0.0. Accordingly, in keeping with one standard practice, we add ½ of a False Positive error to each False Positive count (Hautus et al., 2021).

A one-way ANOVA shows a main effect of condition on *d*′ (*F*(3, 2076) = 6.461, *p* = 0.0002, partial eta-sq = 0.01), and Šídák's multiple comparisons test shows that performance on skin cancer detection task is modestly better in the high prevalence, feedback condition than in the high prevalence, no-feedback condition (*p* = 0.008). Prevalence effects are better understood as criterion shifts. An ANOVA shows a very large main effect of condition on criterion (*F*(3, 2076) = 30.16, *p* < 0.0001, partial eta-sq = 0.04). Pairwise comparisons show a strong LPE effect when feedback is given (*p* < 0.0001). Criterion becomes more conservative at low prevalence. Without feedback, criterion becomes slightly more liberal on average (0.34 to 0.31). This is not statistically significant (*p* = 0.47). As noted earlier, the PICC effect (Levari et al., 2018), while certainly real, is typically more fragile than the LPE effect in the opposite direction (Lyu et al., 2021). The differences between conditions with feedback and without feedback are highly significant (*p* < 0.0001 for each comparison). A significant LPE effect is seen if analysis is restricted to each participant's first block of data (*p* < 0.0001). There is no PICC effect. If all blocks of data are used, eliminating the *d*′ > 0.5 filter, there is again a significant LPE (*p* < 0.0001) and an insignificant PICC effect (*p* = 0.1244).

**Fig. 3:**
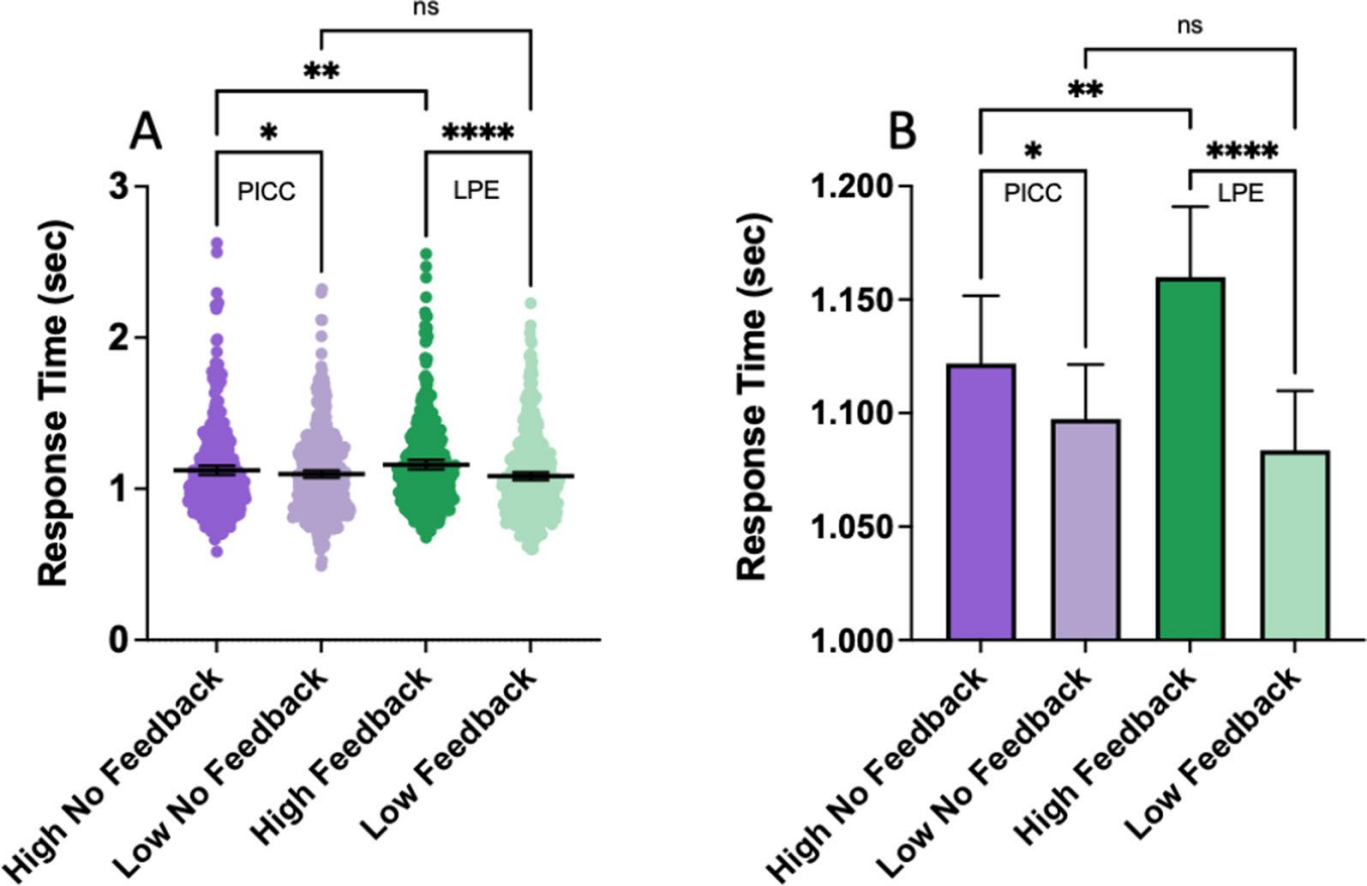
Median response time as a function of block type. RTs < 11 s were analyzed. **A** RT distributions with the graph truncated at 3 s for display purposes. Black lines show the mean. **B** Means of the median RTs on a much finer scale. Error bars show ± 95% confidence intervals

Even though participants were not focused on the speed of their responses, response time data reflect the effects of prevalence and feedback. Figure 3A shows the distribution of the median RTs, divided by block type with one data point per observer per block type. Figure 3B shows the mean and 95% confidence intervals on a much finer scale so that the differences between conditions are more clearly visible. There is a significant effect of block type (*F*(2.889, 978.4) = 15.29, *p* < 0.0001, partial eta-sq = 0.04). Šídák's multiple comparisons test shows that the 76 ms difference between high and low prevalence with feedback is significant (*p* < 0.0001). Low prevalence RTs are shorter than high prevalence. Without feedback,the RT difference between high and low prevalence is much smaller (24 ms, *p* < 0.05). The finding that low prevalence RTs are faster than high prevalence is in line with the RT effects seen in other prevalence studies (Wolfe & VanWert, 2010). This analysis was done with one data point per observer per condition. However, some observers ran multiple sessions of the same condition. If each block is included separately, the difference between high and low prevalence with feedback remains significant, but the difference between high and low prevalence without feedback becomes insignificant. Thus, the no feedback effects should be considered fragile.

### How does one block influence the next?

The block data essentially replicate previous results, collected under very different conditions. However, the key question for this study is whether or not exposure to one combination of prevalence and feedback influences the next block of trials. Blocks could be independent of each other. The effect of one block might have a transient impact on the next block (e.g., lasting for a few trials and then fading). Finally, the effect of one block on the next could be dependent on the delay between blocks (especially since that delay could be from one day to the next). To assess each of these effects, we derive measures of *d*′ and criterion for each of the 16 pairs (combinations of one of four ‘first blocks’ with one of four ‘second blocks’). We then subtract a baseline derived from all blocks of the second block condition. We use the second blocks as the baseline because we are looking for a change in the second block. In fact, it doesn’t make much difference. Many second blocks in one pair are the first blocks in a subsequent pair and the baselines would be very nearly the same if all blocks were included as shown in Fig. 2 above. Thus, for example, to assess the influence on criterion of low prevalence without feedback (condition 1 in the figures to follow) on high prevalence without feedback (cond 3), we compute criterion (pair 13)–criterion (all cond 3); that is, the value for condition 3, when it follow condition 1 minus the value for condition 3, in all settings. This is done for each of the 2080 pairs of blocks using the *d*′ > 0.5 filter. The data for each of these 2080 "good" pairs of blocks are posted on the Open Science Framework at https://osf.io/hck5n/.

**Fig. 4:**
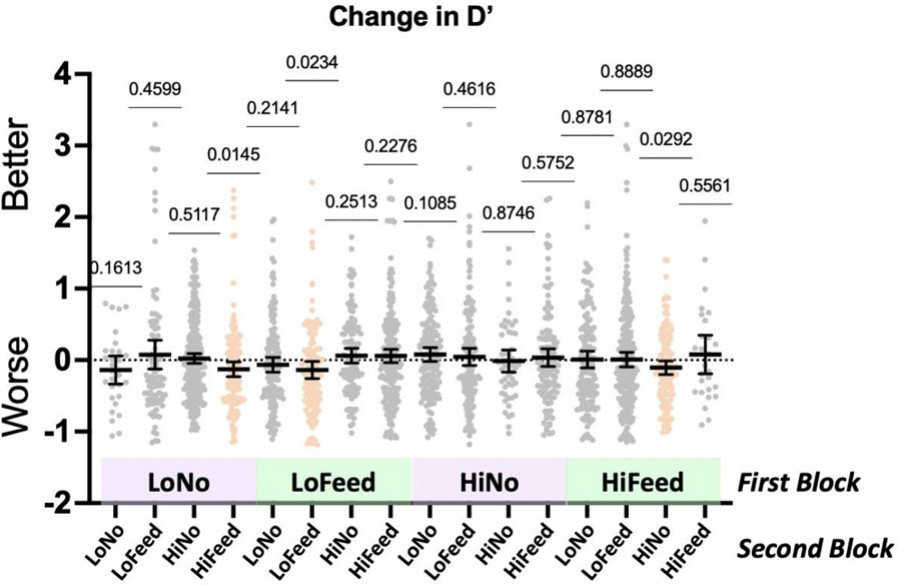
Change in *d*′ on block 2 as a function of the nature of block 1. *p* values show results for simple *t*-tests, testing against the null hypothesis that there is no change in *d*′. Black lines show means ± 95% confidence intervals

Figure 4 shows the change in *D′* as a function of pair with each dot representing one pair of blocks. Statistical tests are simple *T*-tests against a null hypothesis of no change in *d*′ from block 1 to block 2. Three pairs, forming no obvious pattern, reach statistical significance (all *p* =  ~ 0.02, all Cohen's *d* =  ~ 0.2). As these are not corrected for multiple comparisons, these should not be considered strong effects. The general picture is of little or no effect of the first block of trials on *d*′ in the second block of trials.

**Fig. 5:**
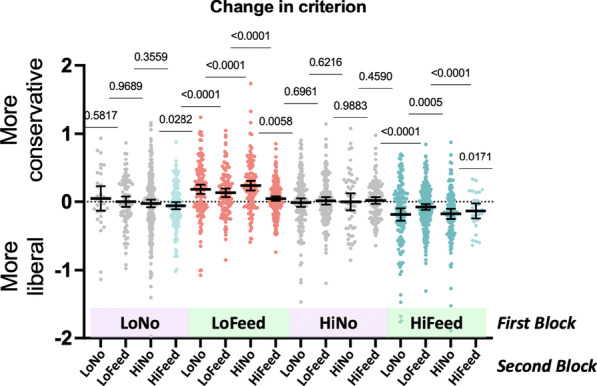
Change in criterion on block 2 as a function of the nature of block 1. *P* values show results for simple *t*-tests, testing against the null hypothesis that there is no change in criterion. Black lines show means ± 95% confidence intervals

A different pattern is seen in the criterion data, plotted in Fig. 5. Here, there are two clear clusters of significant effects. When block 1 has low prevalence and feedback, criterion on block 2 is more conservative (Pairs where Block 1 is low prevalence with feedback, all *p* < 0.006, Cohen's *d* between 0.2 and 0.5). When block 1 has high prevalence and feedback, criterion on block 2 is more liberal (Pairs where Block 1 is high prevalence with feedback, *p* < 0.0005, pair 44, *p* = 0.017; note that there are only 27 pairs of this variety in the dataset, All Cohen's *d* between 0.2 and 0.5). When block 1 does not have feedback, there is no effect on criterion in pair 2, except, perhaps, for ‘high feedback’ on Block 2 (*p* < 0.028, again, not corrected for multiple comparisons, Cohen's *d* = 0.18). This is the main finding of the paper. Criterion can be manipulated by the prevalence of the preceding block of trials, but only if the participants received feedback on that first block.

Looking separately at changes in *P*(True Positive) and *P*(False Positive) responses, we see essentially the same pattern of results. For changes in True Positive proportions, pairs where Block 1 is low prevalence with feedback are significant (all *p* < 0.005) except when Block 2 is High Feedback (*p* = 0.38). Pairs where Block 1 is high prevalence with feedback are significant (all *p* < 0.005 except when Block 2 is Low Feedback which *p* = 0.02). The low feedback–high feedback pair is not significant. For changes in False Positive proportions, pairs where Block 1 is low prevalence with feedback are significant (all *p* < 0.05). When Block 1 is high prevalence with feedback, results are significant when Block 2 is low no feedback, high no feedback, and low feedback (*p* < 0.01). When Block 2 is high feedback, there is no significant change (*p* = 0.29). The low no feedback–high feedback pair is significant (*p* = 0.001). No other changes in *P*(True Positive) and *P*(False Positive) responses are significant. As noted above, removing the *d*′ > 0.5 filter does not change the pattern of results. Limiting analysis to only the first pair of blocks for each participant preserves the direction of effects, but some effects become statistically unreliable because of the loss of power.

### Do the effects of one block on the next change with time between blocks?

**Fig. 6:**
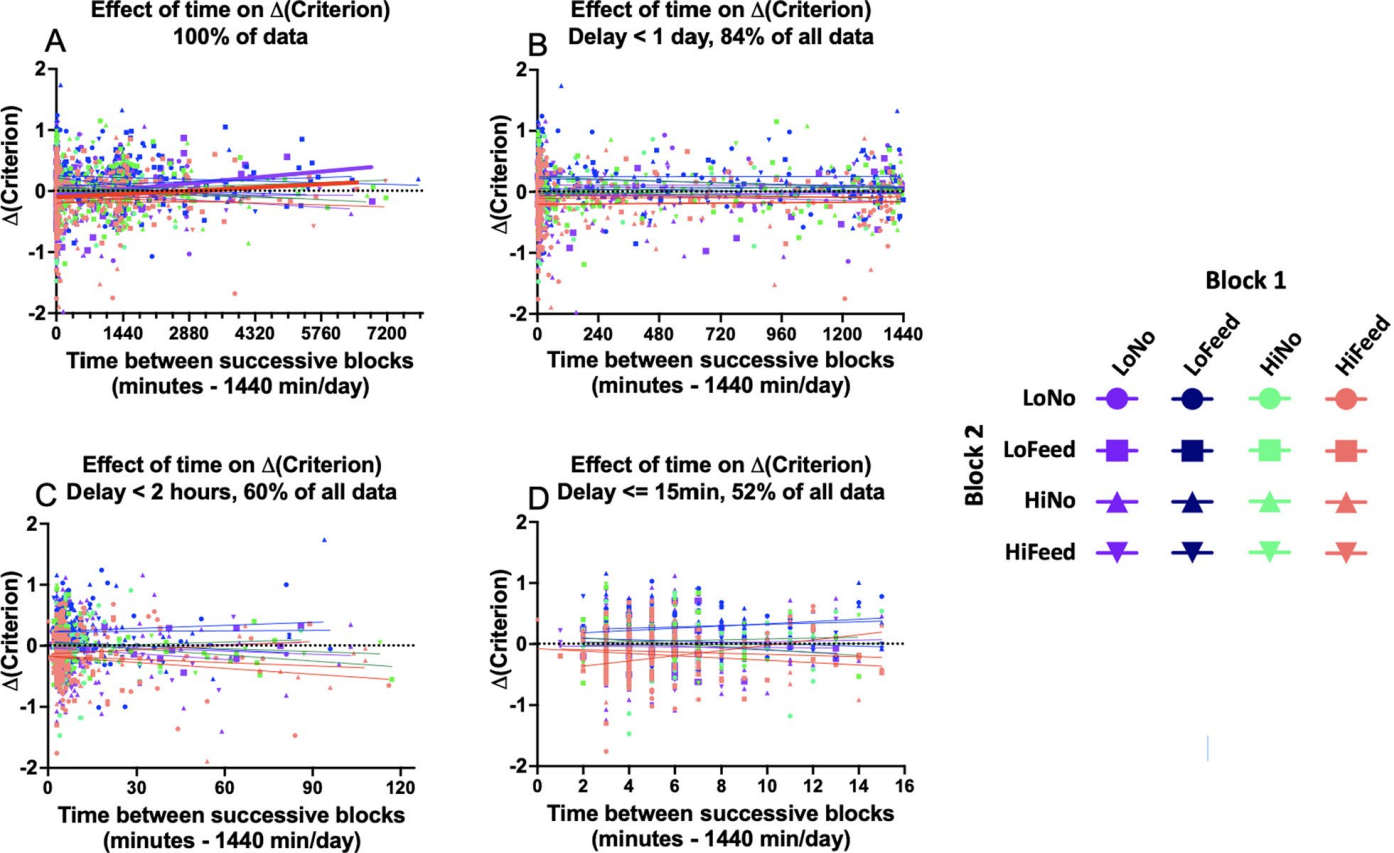
Change in criterion as a function of time between the start of block 1 and start of block 2. 1440 min = 1 day. Colored lines are best-fit linear regressions for each of 16 pairs of block types. **A** All data, **B** Delay < 1 day, **C** Delay < 2 h, **D** Delay < 15 min. Repeat pairs (e.g., Low Feedback → Low Feedback) do not occur for shorter delays (**C**, **D**)

Figure 5 shows that a block with feedback has an influence on the criterion in the next block. Is that effect transitory or persistent? The time between blocks is quite variable because participants could perform the second block immediately or from one to several days later. Accordingly, for each of the 16 pairs of blocks, we examined change in criterion as a function of time between blocks. The results are shown for four different time ranges in Fig. 6.

Because the distribution of delays is strongly skewed toward shorter delays, we repeated the regression analysis separately for the whole dataset, for all delays < 1 day (84% of all data), < 2 h (60% of all data), and 15 min or less (still include 52% of all data because participants tended to do one block and then another, immediately). The four panels of Fig. 6 zoom in to smaller and smaller time scales but, in fact, it does not matter. The point of the admittedly noisy Fig. 6 is that there is no obvious pattern of slopes. This is borne out by linear regressions for each of the 16 pairs of block types. For the entire data set (Fig. 6A), fourteen of sixteen correlations are not significant (all 14 *r*-sq < 0.02, all *p* > 0.16). The low no feedback → low feedback pair has a *r*-sq of 0.06 (*p* = 0.015). The high feedback → low feedback pair has a *r*-sq of 0.02 (*p* = 0.045). The regression lines for those conditions are shown as thicker lines in Fig. 6A. Examining this relationship, the change in criterion is near zero at short times and the change grows more conservative with time. For delays less than 1 day, no correlations are significant (all *r*-sq < 0.02, all *p* > 0.35). For delays less than 2 h, no correlations are significant (all *r*-sq < 0.02, all *p* > 0.20). For delays less than or equal to 15 min, no correlations are significant (all *r*-sq < 0.025, all *p* > 0.24). In sum, there is no evidence for the effects, shown in Fig. 4, fading with time. The two (out of 64) significant correlations seem likely to be random fluctuations of the data and go in the 'wrong' direction if the hypothesis under test is that block 1 would influence block 2 if block 2 occurred recently but not after a longer delay.

**Fig. 7:**
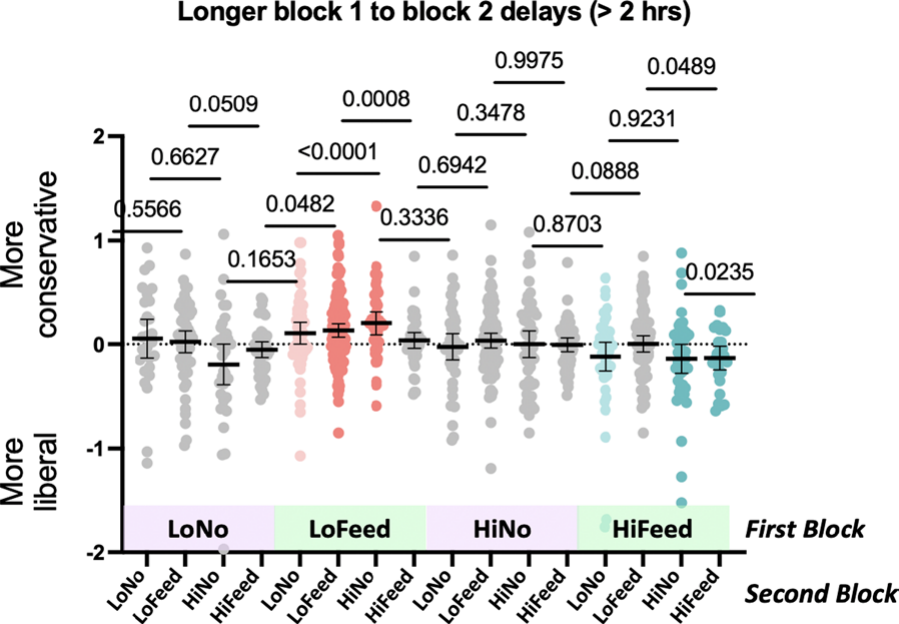
Change in criterion on block 2 as a function of the nature of block 1. Data restricted to pairs separated by more than 2 h

This result is somewhat surprising since, surely, the impact of one block on the next must fade at some point. The results, shown in Fig. 6, tell us that the fading is not fast. In this, the effect of one block seems more like education than adaptation. The first block in the pair is teaching participants something and, like learning the capital of Sweden, that knowledge does not simply fade away within minutes or hours. One might object that the results shown in Fig. 6 are, essentially, negative. As a different way to show that the effects are persistent, Fig. 7 replicates Fig. 5, but with only 40% of the data included, the 40% with block 1–block 2 delays longer than 2 h. The effects get somewhat weaker since most of the data has been discarded, but it is clear that the pattern remains the same. Exposure to a block of low prevalence with feedback makes participants more conservative on the next block. Exposure to a block of high prevalence with feedback makes participants more conservative.

### Does the effect of one block last throughout the next block?

Wolfe and VanWert (2010) did visual search experiments in which target prevalence changed smoothly over 1000 trials. Criterion tracked the change in prevalence with a lag that suggested that criterion was based on the last 2–3 dozen trials. Thus, in an 80-trial block, one might expect the effect of block 1 to be present in the first 40 trials and, perhaps, reduced or absent in the second 40 trials. To assess that possibility, the analysis, shown in Fig. 5, was repeated for the first half and second half of the trials in block 2, separately. The results are essentially unchanged from those shown in Fig. 5. Exposure to low prevalence with feedback in block 1 produces a conservative shift in block 2 criterion in both the first half and the second half of block 2. All *t*-tests for pairs where Block 1 is Low Feedback are significant except for the low feedback → high feedback pair in the first half, when the effect should be stronger than in the second half. Exposure to High Prevalence with Feedback in block 1 produces a liberal shift in block 2 criterion in both the first half and the second half of block 2. *T*-tests when block 2 is low feedback, low no feedback and high no feedback are significant. *T*-test when block 2 is high feedback is not significant in either half (again, recall that the high feedback → high feedback pair has the fewest instances in the dataset).

**Fig. 8:**
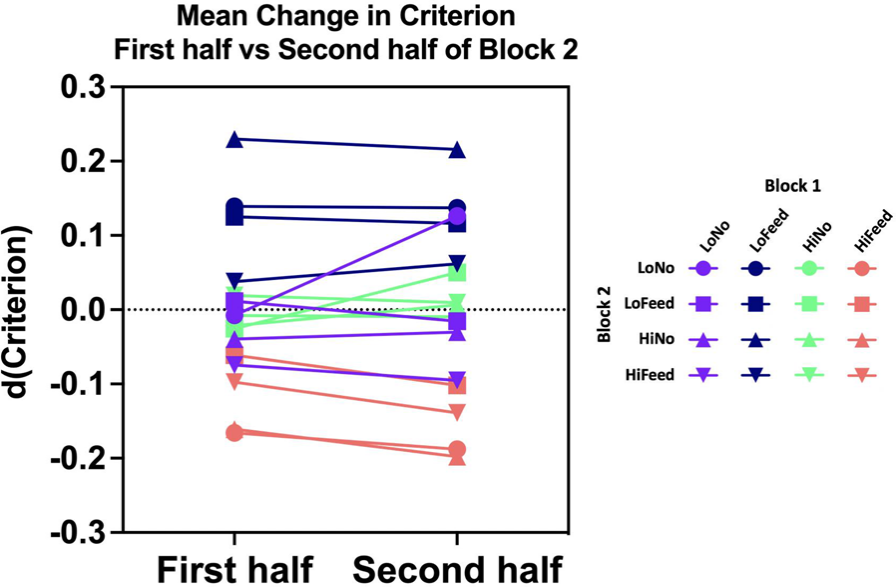
Mean change in criterion for the first and second halves of a block of trials

The lack of any systematic decrease in the magnitude of the effects is shown in Fig. 8. The mean change in criterion is plotted for the first and second half of each pair of blocks. Note the grouping of the pairs. The dark blue pairs show the conservative (positive) shift following low prevalence with feedback. The red pairs show the liberal (negative) shift following high prevalence with feedback. If the effects only lasted for the first 2–3 dozen trials, these effects should collapse toward zero in the second half. Clearly, this is not the case.

### Effects of expertise

As noted in the Methods section, participants gave a rough categorization of their level of expertise. Sadly, this convenience population does not include any substantial number of self-identified expert dermatologists. Only one MD self-identifies as a dermatologist. We can create an expertise continuum from participants reporting no medical training to premedical students to medical students and, finally, to medical doctors. Table 6 shows the numbers of participants and the number of pairs of data contributed by each group.

**Table 6:** Number of participants in each expertise group and the number of condition pairs contributed by the groups

Expertise category	No. of participants	No. of pairs
Medical doctor	54	131
Medical student	337	952
Pre-med student	174	426
No medical experience	145	358

**Fig. 9:**
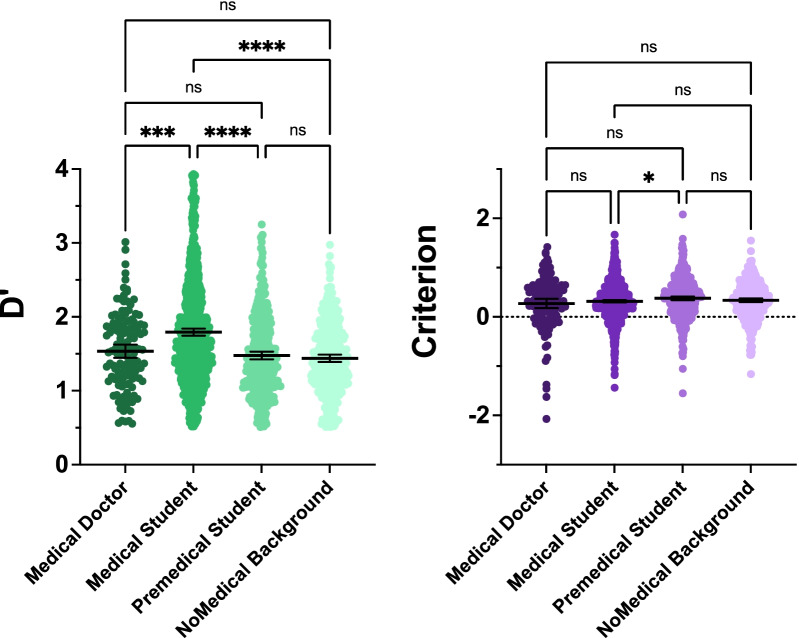
Effects of expertise category on *d*′ and crit, regardless of prevalence and/or feedback

Figure 9 shows *d*′ and criterion as a function of expertise group, regardless of the type of feedback or prevalence (recall, from Fig. 2, that there is little effect of block type on *d*′.)

There is a highly significant effect of expertise category (*F*(3, 1863) = 38.58, *p* < 0.0001, partial eta-squared = 0.86) and, as can be seen by the pairwise comparisons, that effect is entirely due to superior performance of the medical students (all comparisons to medical students, *p* ≤ 0.0001). It might seem disappointing that MDs were no better at this task than non-medical participants. However, these non-dermatologist MDs may not have spent time looking at skin lesions for many years and the novices were only being asked to perform a difficult, but straightforward, two-alternative, forced-choice discrimination. It is the medical students, many of whom may have recently been learning about skin lesions, who do somewhat better on this task. Of course, there could be other reasons for the group differences (e.g., motivated, competitive students versus more casually interested MDs. We cannot know in this case.)

There is a very modest effect of expertise category on criterion (*F*(3, 1863) = 3.32, *p* = 0.02, partial eta-squared = 0.01). Pairwise comparisons reveal that premedical students are slightly more conservative than medical students (*p* = 0.049). This does not appear to be a particularly meaningful finding.

In terms of the impact of expertise on the effect of block 1 on block 2, the data are noisy, with fewer pairs showing significant effects of block 1 on block 2. This seems to be a statistical power issue. The pairs that are significant are the same as those shown in Fig. 4, with low prevalence on block 1 making participants more conservative on block 2 and high prevalence making them more liberal. There is no evidence for any systematic effect of expertise. In particular, there is no evidence that the most expert group (medical students) shows a different pattern of results. In that group (fortunately, the largest), pairs with low feedback as block 1 are more conservative and with high feedback as block 1 are more liberal (all *p* <= 0.01).

## General discussion

To summarize, this large sample of online volunteers replicated prior findings concerning the effects of prevalence. With feedback, low prevalence makes participants more conservative. Without feedback, this classic low prevalence effect (LPE) is not seen and, indeed, it is numerically, if not statistically reversed, to become a weak example of a prevalence-induced concept change (PICC). Thus, the basic pattern of prevalence results can be replicated with a novel stimulus set (skin lesions). We found no evidence that the effects were modulated by expertise (though our measure of expertise is crude). This is consistent with other findings of prevalence effects in expert populations (Evans et al., 2011, 2013; Evered, 2017; Wolfe et al., 2013). Prevalence effects seem to be part of basic human cognition.

Beyond replication, the new information in this dataset concerns the impact of one block of trials on the next. In much of the prior work, high prevalence was followed by low prevalence or prevalence conditions were free standing with different prevalence run on different groups of participants. In this experiment, we were able to look at the effect of each of four conditions in block one on each of those conditions in block two, yielding sixteen different pairs. The results are quite clear. Experiencing a block of low prevalence with feedback makes participants subsequently more conservative. Experiencing a block of high prevalence with feedback makes them more liberal. Blocks without feedback seem to have no measurable impact on the next block. Interestingly, this pattern of results does not appear to be influenced by the delay between block 1 and block 2 (Figs. 6, 7). Nor does the effect appear to wane in the second half of an 80-trial second block (Fig. 8).

**Fig. 10:**
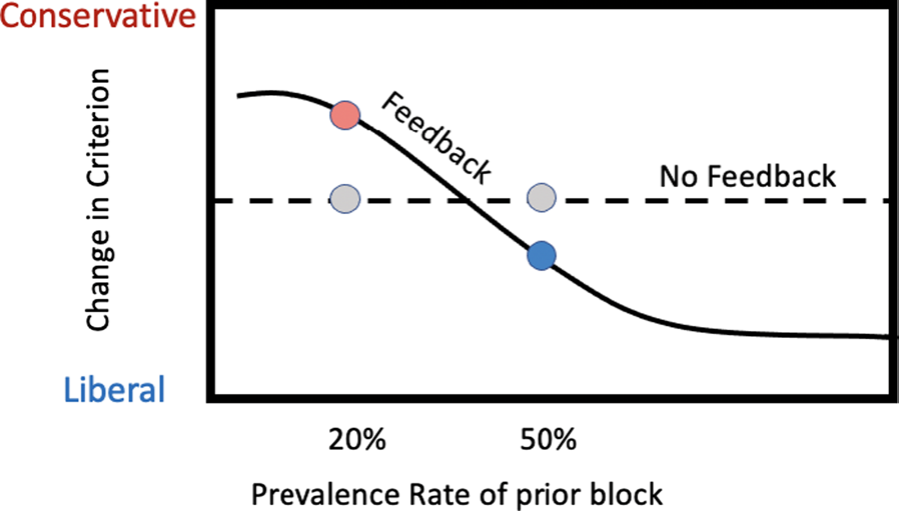
Hypothetical curves showing the results of the feedback and no feedback conditions as two points on continuous functions

Presumably, participants are learning something from the feedback. When target prevalence is low, most of the errors will be false positive errors. If the rate of false positive errors is above what seems right (explicitly, or more likely, implicitly), the response would be to make fewer positive responses. This would be a shift to a more conservative point, resulting in fewer false positives but more false negatives. At 50% prevalence, the number of false positive and false negative errors would be equal at a neutral criterion. Apparently, this does not correspond to what participants, on average, feel is right either. Participants behave as if they are making too many negative responses and shift the average criterion to a more liberal position resulting in fewer false negatives but more false positives. Note that the 50% prevalence feedback block produces the most liberal average criterion as shown in Fig. 2. It is more liberal than 50% without feedback or 20% with feedback on the next block. Exposure to 50% prevalence with feedback makes participants a bit more liberal on the next block, too. This situation is cartooned in Fig. 10 which makes the point that there must be some level of prevalence that, if present on Block 1, would produce no average shift in criterion on Block 2. It would be interesting to map out the hypothetical function shown in Fig. 10 and to try to understand what determines that hypothetical neutral point. The no feedback conditions (shown in gray in Fig. 10) are not 'educational', producing no significant changes in criterion in this experiment, though, in other studies, participants appear to learn from their rate of response, becoming more liberal at low prevalence ("I am not saying 'present' often enough.") and conservative at higher prevalence (Levari et al., 2018; Lyu et al., 2021). But any educational effects of the no-feedback conditions do not appear to transfer to the next block.

Other studies have found evidence that feedback can be used to deliberately manipulate criterion. One approach is to give observers false feedback. In early work on vigilance, Jane Mackworth (1964) found that false feedback produced better performance than no feedback, perhaps by increasing motivation. Schwark et al (2012) found that they could reduce miss errors in a search task by falsely telling observers that they had missed targets. Cox et al. (2021) were able to produce different error rates and criteria by telling observers at the start of a trial either that a display contained "up to two" or "one or two" targets. Observers made more errors with the "up to two targets" instruction. That instruction held out the possibility of target absent trials in an experiment that had no such trials and, as a result, observers sometimes incorrectly concluded that no target was present. Han and Dobbins (2008) found that they could move criterion on a trial by trial basis by incorrectly informing observers either that their false negative responses were correct, which made observers more conservative, or that their false positive responses were correct, making observers more liberal. This is interesting because, as they note, there is a body of previous work suggesting that criterion is resistant to movement based on truthful feedback during the experiment. Criterion can be moved by explicit instructions before the block of trials (e.g., Reed et al., 2014; Littlefair et al., 2016). This makes one wonder if the effects reported here consist of block 1 acting as explicit instructions for block 2. We did not ask participants what they knew about target prevalence or their own criterion, making this a potentially interesting topic for future research.

Thinking about the effect of block 1 on block 2 as 'education' raises the possibility of using this knowledge in the real world. Specifically, it raises the possibility that the prevalence used during training (presumably with feedback) could have long-term effects on criterion once training is over, especially if post-training feedback is limited, since the no-feedback conditions move criterion less vigorously. One step to determining if there is a real-world application of these findings would be to determine how widely they generalize. In this paper, we have shown an effect of one block of trials on a very similar subsequent block of trials. Would we see the same effects if the setting changed: e.g., to a situation where block 1 was clearly part of training and block 2 was the task in the field? Would the effect of block 1 influence a second block that used different stimuli or a different task: e.g., if block 1 trained on a 2AFC cancer/not cancer discrimination while block 2 involved a *search* for cancer in a screening setting such as mammographic breast cancer screening. Our data failed to show any decrease in the effect of block 1 after a day or after the first 40 trials of the next block, but one would like to know if something like an 80-trial, block 1 would produce an effect on the rest of the day's work in the real world.

### Limitations

While the results of this study appear to be quite robust and straight-forward, the study does have several short-comings, mostly due to the nature of the convenience sample we used. With 803 participants, we do seem to have good statistical power, even after various exclusions, discussed above. However, the number of block 1–block 2 pairs where the two blocks are identical is reduced because participants could only run one block of a given condition each day. This task structure also means that all such identical pairs are separated by at least a day while other blocks could be run within minutes of each other. Different participants contribute different numbers of blocks and pairs. In a perfect experimental world, each observer would contribute the same number of pairs and these would be counterbalanced for order effect, etc. It would be difficult to carry out such an experiment with a large group of participants. When we filter the data in a posthoc manner (e.g., to have a single pair from each observer), we obtain a similar pattern of results to those shown here, but we lose statistical power. Thus, we are quite confident in the main findings of the paper, even if the design is unbalanced.

Given the online nature of the study, we lack control over the display and ambient lighting. It seems likely that *d*′ would improve if we optimized viewing conditions. It is not obvious why there might be a marked effect on criterion, but it is possible. Finally, for a study of skin cancer images, we lack a population of true dermatology experts. It would be worth trying to re-run at least a subset of the 16 pairs on a population of experts who, one may hope, would have *d*′ values comfortably above those of medical students.

Finally, there are other possible analyses that could be performed (and the data are posted at https://osf.io/hck5n/). For instance, there could be interesting trial-by-trial results akin to those of Fischer and Whitney (2014)

### Conclusion

The primary conclusion of this paper is that feedback educates observers, causing them to become more liberal when targets have been relatively common and more conservative when those targets are rare. The effects of a block of trials with feedback can last for days with those effects showing up when the observer takes up a similar task again. It may be possible to use the educational effects of feedback when it is desirable to shift an observer's criterion, especially if the subsequent task does not involve reliable feedback.

## Data Availability

Excel spreadsheets with all data and basic analyses are posted on the Open Science Framework at https://osf.io/hck5n/.

## Abbreviations

LPE: Low prevalence effect
PICC: Prevalence-induced concept change
